# Correlation between Potential Risk Factors and Pulmonary Embolism in Sarcoidosis Patients Timely Treated

**DOI:** 10.3390/jcm10112462

**Published:** 2021-06-02

**Authors:** Barbara Ruaro, Paola Confalonieri, Mario Santagiuliana, Barbara Wade, Elisa Baratella, Metka Kodric, Maria Berria, Mohamad Jaber, Chiara Torregiani, Cosimo Bruni, Marco Confalonieri, Francesco Salton

**Affiliations:** 1 Department of Pulmonology, University Hospital of Cattinara, 34127 Trieste, Italy; paola.confalonieri.24@gmail.com (P.C.); mario@marionline.it (M.S.); metka.kodric@asuits.sanita.fvg.it (M.K.); mohamad.jaber@asugi.sanita.fvg.it (M.J.); torricina@gmail.com (C.T.); marco.confalonieri@asugi.sanita.fvg.it (M.C.); francesco.salton@gmail.com (F.S.); 2 AOU City of Health and Science of Turin, Department of Science of Public Health and Pediatrics, University of Torino, 10126 Torino, Italy; barbarawade@hotmail.com; 3 Department of Radiology, Cattinara Hospital, University of Trieste, 34127 Trieste, Italy; elisa.baratella@gmail.com; 4 Department of Internal Medicine, University of Sassari, 07100 Sassari, Italy; mariaberria@icloud.com; 5 Department of Experimental and Clinical Medicine, Division of Rheumatology, University of Firenze, 50121 Florence, Italy; cosimobruni85@gmail.com

**Keywords:** sarcoidosis, pulmonary embolism, scintigraphy, antiphospholipid antibodies, timely treatment

## Abstract

Background. Some studies with inconclusive results have reported a link between sarcoidosis and an increased risk of pulmonary embolism (PE). This study aimed at assessing a possible correlation between potential risk factors and PE in sarcoidosis patients. Methods. A total of 256 sarcoidosis patients (84 males and 172 females; mean age at diagnosis 49 ± 13) were enrolled after giving written informed consent. Clinical evaluations, laboratory and radiology tests were performed to evaluate the presence of pulmonary embolism. Results. Fifteen sarcoidosis patients with PE (4 males and 11 females; mean age at diagnosis 50 ± 11), diagnosed by lung scintigraphy and 241 sarcoidosis patients without PE (80 males and 161 females; mean age at diagnosis 47 ± 13), were observed. There was a statistically significant increase of the presence of antiphospholipid antibodies in the sarcoidosis group with pulmonary embolism. There was no statistically significant difference between the two groups as to smoking habit, obesity or hereditary thrombophilia frequency (*p* > 0.05, respectively). Conclusions. This study demonstrates a significant correlation between the presence of antiphospholipid antibody positivity and the pulmonary embolism events in our sarcoidosis patients. Furthermore, we propose screening for these antibodies and monitoring, aimed at timely treatment.

## 1. Introduction

Sarcoidosis is a frequently insidious, rare, multiorgan disease, and diagnosis is often a chance finding at a routine chest radiography, carried out for other reasons, typically observed in adolescents or young adults [1,2]. Lung involvement is common and symptoms may include cough, dyspnea and chest pain. Extrapulmonary symptoms may involve the skin, joints and eyes. The chest radiograph typically evidence bilateral hilar adenopathy [1,2,3,4], and although there may be a rise in angiotensin converting enzyme levels, this finding is not diagnostic and histopathological confirmation of noncaseating granulomas is required to confirm diagnosis [2,3,4,5,6,7,8,9,10,11]. This biopsy is usually obtained from the most peripheral site possible, although transbronchial biopsy is commonly required [1,2,3,4,5,6].

The increased risk of pulmonary embolism (PE) in sarcoidosis patients is a relatively recent finding [12,13,14,15,16,17]. Indeed, there has been a progressive increase in the knowledge of the pathology and pathogenetic mechanisms involved in sarcoidosis, which has led to the observation of this association. However, to date, the actual incidence or co-incidence of PE and sarcoidosis remains unknown. Whether sarcoidosis represents a “distinct” risk factor for PE has not yet been fully defined and further studies will hopefully clarify this point, as the currently available literature is scarce [16,17,18,19,20].

The primary aim of this study was to evaluate the frequency of PE events in the sarcoidosis patients attending our national referral center for the disease. The secondary objective was to determine possible clinical sarcoidosis phenotype correlate with PE complications.

## 2. Materials and Methods

### 2.1. Patient Population

After having given written informed consent, a total of 256 patients affected by sarcoidosis (134 males and 122 females; mean age at diagnosis 49 ± 13) were enrolled. Diagnosis was made during a routine clinical assessment in our pulmonology department, referral center for sarcoidosis, from January, 2020 to December, 2020 [3,4,10]. A complete medical history was collected, and all patients were examined clinically. Demographic and clinical data (i.e., age, gender, smoking status, obesity defined as body mass index—BMI > 30, treatment regimens) were also recorded (see Table 1). We excluded subjects with insufficient clinical information (i.e., age at diagnosis, smoking habit), missing blood tests or diagnostic imaging results, active cancer, connective tissue diseases and surgical intervention or immobilization in the last month. The exclusion criteria for lung embolism were: a normal perfusion pattern at scintigraphy; perfusion defects not arranged with the lung vessels and CT-visualized perfusion defects caused by abnormalities in the lung parenchyma.

Blood tests (detailed in Section 2.2), pulmonary function tests (detailed in Section 2.3) and imaging evaluations (detailed in Section 2.4) were performed for all patients (see Table 2).

### 2.2. Blood Tests

After obtaining written informed consent, a complete blood chemistry evaluation was made, i.e., a total blood count, the CD4/CD8 lymphocyte ratio, angiotensin converting enzyme (ACE), D-dimer level, antiphospholipid antibodies (antibodies anti-cardiolipin, antibodies anti-beta2-glycoprotein, lupus anticoagulant), thrombophilic screening (Factor V Leiden alteration, protein C deficiency, protein S deficiency, factor II mutation, hyperhomocysteinemia/MTHFR gene mutation).

### 2.3. Pulmonary Function Tests

Pulmonary function tests (PFTs) are essential, readily available, and non-invasive tests. The PFTs were performed in the Pulmonology Units of the University Hospital of Trieste, the same operator and equipment evaluated all patients. Global spirometry values were recorded, i.e., functional vital capacity (FVC), forced expiratory volume in 1 s (FEV1), forced expiratory volume in 1 s/functional vital capacity (FEV1/FVC) ratio, also known as the Tiffenau Index (IT) and diffusion capacity of carbon monoxide (DLCO).

### 2.4. Lung Scintigraphy

All patients with a clinical or laboratory suspicion of pulmonary embolism (PE) were evaluated by scintigraphy, i.e., the most common evaluation method for the diagnosis of PE.

Pulmonary perfusion assessment was performed after an intravenous injection of albumin macroaggregates labelled with radioactive technetium (^99m^Tc-MAA Makro-Albumon^®^; Medi-Radiopharma Ltd., Érd, Hungary) intravenous. The imaging procedure was commenced immediately after the administration of the radiotracer and the images were acquired by a SPECT/CT hybrid dual-head gamma camera, Infinia VC Hawkeye 4 (GE Healthcare, General Electric Healthcare, Chicago, IL, USA). All SPECT/CT acquisitions were obtained with the patient in the same position. The SPECT/CT images were interpreted by Xeleris 1 and 2 Functional Imaging Workstations (GE Healthcare). The scintigrams were analyzed by two expert nuclear medicine specialists (with 7 and 28 years of experience, respectively).

Based on the SPECT/CT results, the following criteria for the diagnosis of PE were adopted: at least 1 segmental or two sub-segmental perfusion defects (wedge-shaped, base directed towards the pleura) without corresponding abnormalities in the pulmonary parenchyma.

### 2.5. Statistical Analysis

Continuous variables were summarized by mean and standard deviation (SD). Student’s *t*-test and the Mann-Whitney test assessed continuous variables, while categorical variables were compared by the Chi-Square test of independence or by Fischer’s exact test, when appropriate. The data analysis was made by the Software R (Free Software/Open Source, Lucent Technologies, Murray Hill, NJ, USA; version 4.0.2, 2020). The level of statistical significance was set at a *p*-value of < 0.05.

## 3. Results

The study population included 256 patients affected by sarcoidosis (84 males and 172 females; mean age at diagnosis 49 ± 13) who were enrolled over a 1-year period after having given written informed consent (see Table 1). A total of 15 sarcoidosis patients with PE (4 males and 11 females; mean age at diagnosis 50 ± 11) were observed in our study population and 241 sarcoidosis patients without PE (80 males and 161 females; mean age at diagnosis 47 ± 13).

No statistical difference was observed in the age at onset of the disease and/or at enrollment (Table 1). Despite the prevalence of female sex in both groups (73% of females in group with PE group and 67% in group without PE), this datum did not reach statistical significance. The percentages of current and former smokers were similar among groups, as were the percentage of obesity and the therapy regimens. There was no significant difference between the two groups, with or without PE, regarding cardiac comorbidities (e.g., arterial hypertension, arrhythmias, valve diseases), immunological disorders (e.g., Basedow’s disease, Hashimoto’s thyroiditis, vitiligo) and history of cancer. However, there was a statistically significant difference in the number of previous episodes of venous thromboembolism.

The analysis of the two groups of patients (Table 2) showed that there was no statistically significant difference in the percentage of sarcoidosis organ involvement, laboratory tests (including the ACE concentration and thrombophilic parameters) and in the PFTs. Conversely, a statistically significant difference in the presence of antiphospholipid antibodies was observed between sarcoidosis patients with and without PE. Three of the patients with PE had increased levels of IgG antibodies anticardiolipin, two had IgG antibodies anti-beta2-glycoprotein, and two lupus anticoagulant positivity.

None of our patients with pulmonary embolism had hemodynamic instability requiring admission to the intensive care unit for thrombolytic therapy. All patients were treated by oral anticoagulant therapy. Regarding the outcome, all PE patients survived, also because they were treated in a timely fashion. Both groups are continuing their follow-up. To the best of our knowledge today, there have been no new cases of PE in the two groups.

## 4. Discussion

The study confirmed an increase in PE in our sarcoidosis patients (5.85% of our study population) [18,19]. This rate is significantly higher than expected in the general population without sarcoidosis. Indeed, the incidence of PE in the general population is estimated to be around 60 to 70 per 100,000, and that of venous thrombosis approximately 124 per 100,000 [18,19]. Furthermore, there was an increased risk of PE in patients with previous episodes of venous thromboembolism, a reduced blood ratio between CD4 and CD8 lymphocytes and antiphospholipid antibody positivity. A recent Swedish survey, based on the Swedish National Patient Register and The Cause of Death Register, reported a hazard ratio for pulmonary embolism of 4.36 (95% CI 2.26–7.07) in 7828 sarcoidosis patients compared to 15,656 controls, from 2007 to 2016 [20]. A Swedish study and a previous large meta-analysis [12,20] reported a higher incidence of malignant neoplasms in the sarcoidosis patients than in controls, which may, in part, account for the higher incidence of PE in sarcoidosis. Nevertheless, the finding of PE was unrelated to the presence of malignancies in our study population.

Our study population was in line with several other studies regarding the prevalence of females and age of disease onset [12,20]. Swigris et al. evaluated death certificates from 1988 to 2007 and observed 46,450,489 deaths in the United States (US) and 23,679 decedents with sarcoidosis [12]. The presence of PE was observed on 2.54% of the patients’ death certificates, which corresponds to 1.13% of the background population. That is, they found an increased risk of PE among US decedents with sarcoidosis [12].

Vorselaars et al. also observed a high number of PE (6.2%) in their sarcoidosis patients, emphasizing that this percentage was even higher than the 2.5% reported by Swigris et al., stating that this discrepancy may be due to the percentage of PE decedents with sarcoidosis and that the percentage of PE in a consecutive cohort of patients with sarcoidosis cannot be compared accurately [8,12].

Another study, by Crawshaw et al., reported a two-fold higher PE rate in sarcoidosis patients in a 35-year record linkage study [13].

However, the underlying cause of the association between sarcoidosis and PE remains speculative [21,22,23,24,25,26,27,28,29,30,31,32,33,34,35,36]. Some authors have hypothesized a role for medication (e.g., corticosteroid and immunosuppressive drugs) [8,24,25,26]. This finding differed from what was observed in our study, as no statistically significant difference was observed in the percentage of drugs taken by PE and non-PE sarcoidosis patients. Our findings as to the smoking habit not being a well-established risk factor for PE is in line with other authors [8,12,20,24].

Other studies have reported that sarcoidosis, as with other chronic inflammatory conditions, is associated with an increased risk of venous thromboembolism (VTE) [5,12,21,22]. Several studies hypothesize that chronic inflammation in association with sarcoidosis may well predispose to endothelial cell injury, with the inflammatory cytokines activating the coagulation cascade and that the reduced ratio between CD4 and CD8 lymphocytes is likely to be involved in this process [19,20,21,22,23,24,25,26,27,28]. Several studies have reported that lymphocytes are depleted in peripheral blood due to increased infiltration of target organs. However, the same studies observed that in bronchoalveolar lavage (BAL), there was a significant increase in the number of CD4 positive T cells in the BAL fluid and a decrease in the absolute number of CD4 positive T cells in the peripheral blood [35,36].

Few studies have reported on antiphospholipid syndrome and their findings differed with respect to the presence of antiphospholipid antibodies (38% vs. 2–5%) in sarcoidosis patients [1,2,3,8,13,31,32,33]. It has been reported in the literature that the presence of antiphospholipid antibodies in sarcoidosis patients is correlated with a poor prognosis [8,13,31,32,33], and some authors state that these antibodies are disease markers with a prolonged course and are associated with extrathoracic involvement and persistence of radiographic changes [30,31,32,33,34]. It is known that antiphospholipid antibodies are risk factors for venous thromboembolism and their positivity predisposes to the development of thrombotic complications, even if this positivity does not always determine clinical manifestations. The clinical relevance of positivity for these antibodies must take into account the type of positivity among those tested, the antibody titer, the persistence of positivity and the number of determinations where they are positive [30,31,32,33,34]. In our study, none of the subjects were diagnosed with antiphospholipid antibody syndrome. These data are in agreement with what has been reported in the literature, where this association has rarely been found [33,34].

Although this observational study has limitations, e.g., the single-center study design and the presence of Caucasian patients only, the study population was in line with several other studies as to the prevalence of females and age of disease onset [12,20]. Finally, we did not perform a multivariable analysis in order to find independent predictors of PE.

## 5. Conclusions

In conclusion, we suggest that sarcoidosis patients should be closely monitored for: (a) the presence of antiphospholipid antibody positivity; (b) any previous venous thromboembolism episodes; and (c) a reduced CD4 and CD8 lymphocyte ratio. Indeed, all patients with sarcoidosis and/or worsening of respiratory function, especially those with severe and chronic disease, should be carefully investigated for the presence of PE, as it is a potentially fatal complication and a cause of dyspnea. Furthermore, the data herein reported support the need for further prospective studies into the underlying causes and relative risk of PE in sarcoidosis patients.

## Figures and Tables

**Table 1:** Clinical characteristics of the study population at enrollment.

Variables	Sarcoidosis Patients with PE (*n* = 15)	Sarcoidosis Patients without PE (*n* = 241)	*p*-Value
**Clinical Characteristics**			
Gender (*n*, %) Male; Female	M: 4 (27%); F: 11 (73%)	M: 80 (33%) F: 161 (67%)	*p* > 0.05
Age at diagnosis (mean ± SD)	50 ± 11	47 ± 13	*p* > 0.05
Age at enrollment (mean ± SD)	55 ± 12	54 ± 13	*p* > 0.05
Current smoker (*n*, %)	1 (7%)	14 (6%)	*p* > 0.05
Former smoker (*n*, %)	2 (13%)	35 (14%)	*p* > 0.05
Obesity, BMI > 30 (*n*, %)	2 (13%)	27 (11%)	*p* > 0.05
Previous episodes of venous thromboembolism (*n*, %)	3 (20%)	8 (3%)	*p* = 0.020
Cardiac comorbidities (*n*, %)	2 (13%)	27 (11%)	*p* > 0.05
Immunological disorders (*n*, %)	2 (13%)	19 (9%)	*p* > 0.05
History of cancers (*n*, %)	1 (7%)	7 (3%)	*p* > 0.05
**Treatment Regimens**			
Corticosteroids (*n*, %)	11 (73%)	190 (78%)	*p* > 0.05
Methotrexate (*n*, %)	7 (46%)	107 (44%)	*p* > 0.05
Hydroxychloroquine (*n*, %)	6 (40%)	80 (34%)	*p* > 0.05
Biological drugs (*n*, %)	1 (7%)	8 (3%)	*p* > 0.05
Pentoxifylline (*n*, %)	2 (13%)	34 (14%)	*p* > 0.05
Mycophenolate mofetil (*n*, %)	0 (0%)	3 (1%)	*p* > 0.05
Azathioprine (*n*, %)	1 (7%)	11 (5%)	*p* > 0.05

Legend: BMI = body mass index, *n* = number of patients, % = percentage of patients.

**Table 2:** Characteristics of the study population at enrollment.

Variables	Sarcoidosis Patients with PE (*n* = 15)	Sarcoidosis Patients without PE (*n* = 241)	*p*-Value
**Sarcoidosis Organ Involvement**			
Lymph node involvement (*n*, %)	13 (87%)	205 (85%)	*p* > 0.05
Lung (*n*, %)	11 (73%)	187 (77%)	*p* > 0.05
Renal/Hypercalciuria (*n*, %)	5 (33%)	74 (31%)	*p* > 0.05
Cutaneous (*n*, %)	4 (26%)	54 (22%)	*p* > 0.05
Osteoarticular (*n*, %)	2 (13%)	36 (15%)	*p* > 0.05
Abdominal (*n*, %)	2 (13%)	25 (10%)	*p* > 0.05
Cardiac (*n*, %)	0 (0%)	9 (3%)	*p* > 0.05
Neurological (*n*, %)	1 (7%)	7 (3%)	*p* > 0.05
Ocular (*n*, %)	1 (7%)	7 (3%)	*p* > 0.05
Lofgren’s syndrome (*n*, %)	1 (7%)	5 (2%)	*p* > 0.05
**Blood Tests**			
ACE (U/L) (mean ± SD)	43.73 ± 45.17	38.92 ± 34.65	*p* > 0.05
D-dimer (500 ng/mL FEU)	279 ± 184	301 ± 162	*p* > 0.05
CD4/CD8 lymphocytes (mean ± SD)	2.06 ± 1.21	3.68 ± 1.33	*p* = 0.013
Thrombophilic screening positivity (*n*, %)	5 (33%)	16 (6%)	*p* > 0.05
Antiphospholipid antibodies positivity (*n*, %)	7 (46%)	23 (9%)	*p* = 0.026
**Pulmonary Function Tests**			
FVC (mean ± SD)	102.78 ± 18.16	104.79 ± 18.03	*p* > 0.05
FEV1 (mean ± SD)	97.07 ± 15.70	96.90 ± 20.17	*p* > 0.05
IT (mean ± SD)	79.57 ± 7.83	76.79 ± 9.33	*p* > 0.05
DLCO (mean ± SD)	88.53 ± 20.43	86.83 ± 21.41	*p* > 0.05

Legend. *n* = number of patients, % = percentage of patients; ACE = angiotensin converting enzyme, FEU = Fibrinogen Equivalent Units, FVC = functional vital capacity, FEV1 = forced expiratory volume in 1 s, IT = Tiffenau index = forced expiratory volume in 1 s/functional vital capacity, DLCO = diffusion capacity of carbon monoxide.

## Data Availability

All the data are available upon reasonable request to the corresponding author.
